# Low progesterone receptor levels in high-grade DCIS correlate with HER2 upregulation and the presence of invasive components

**DOI:** 10.3389/fonc.2024.1347166

**Published:** 2024-06-26

**Authors:** Hossein Schandiz, Lorant Farkas, Daehoon Park, Yan Liu, Solveig N. Andersen, Jürgen Geisler, Torill Sauer

**Affiliations:** ^1^ Department of Oncology, Akershus University Hospital (AHUS), Lorenskog, Norway; ^2^ Institute of Clinical Medicine, Faculty of Medicine, University of Oslo, Oslo, Norway; ^3^ Department of Pathology, Oslo University Hospital, Oslo, Norway; ^4^ Department of Clinical Molecular Biology (EpiGen), Akershus University Hospital (AHUS), Lorenskog, Norway

**Keywords:** ductal carcinoma *in situ*, invasive breast carcinoma, molecular subtypes, precision medicine, immunohistochemistry, hormone receptors, HER2, *in situ* hybridization

## Abstract

**Objective:**

In this study, we investigated pivotal molecular markers in human high-grade breast ductal carcinoma *in situ* (DCIS). Expression status of estrogen receptor (ER), progesterone receptor (PR), and human epidermal growth receptor 2 (HER2) was measured among various subtypes (Luminal (Lum) A, LumB HER2^-^, LumB HER2^+^, HER2-enriched and triple-negative).

**Methods:**

In total, 357 DCIS cases were classified into respective subtypes, according to the 2013 St. Gallen guidelines. Each subtype was categorized into three subcategories: “Pure” (those without an invasive component), “W/invasive” (those with an invasive component), and “All” (the entire group of the given subtype). ER and PR expression were registered as intervals. Equivocal HER2 immunohistochemistry (IHC) cases (2+) were further investigated using dual-color *in situ* hybridization.

**Results:**

The majority of patients (71%) were over the age of 50. We discovered no significant differences in the proportion of age between the “Pure” and “W/invasive” groups. There was no significant difference in ER/PR expression between “Pure” luminal subtypes of DCIS and “W/invasive” cases. We compared the HER2 IHC scores of “0”, “1+”, and “2+” among LumA and LumB HER2 subtypes and identified no statistically significant differences between “Pure” and “W/invasive” (*p* = 0.603). ER and PR expression ≥ 50% cutoff value was present in > 90% of all LumA cases. The incidences of cases with ER expression at cutoff values of < 10% and ≥ 50% in LumA were significantly different compared to other luminal subtypes (*p* < 0.0001). The proportion of cases with PR expression < 20% showed significant differences in the various luminal subtypes. In luminal B subtypes, low PR expression (< 20%) was significantly associated with both strong HER2 expression (3+) and the presence of an invasive component (*p* = 0.0001 and *p* = 0.0365, respectively).

**Conclusions:**

ER and PR expression at ≥ 50% cutoff values were found in more than 90% of LumA cases. Samples with ER < 10% and ≥ 50% in LumA were significantly different compared to other luminal subtypes (*p* < 0.0001). Low PR expression in high-grade DCIS was strongly associated with HER2 overexpression (3+) and an invasive component (*p* = 0.0001 and *p* = 0.0365, respectively).

## Introduction

Breast ductal carcinoma *in situ* (DCIS), a precancerous lesion that is considered to be a precursor of invasive breast carcinoma (IBC), is a frequent finding during modern breast cancer diagnostics after mammography screening was implemented (1). In industrialized countries, the incidence of DCIS is approximately 20%–25% of all malignant lesions detected in national screening programs (1, 2). Only a proportion of these lesions eventually transform into IBC. If left untreated, it is predicted that 8%–17.6% of DCIS cases may progress to IBC after 10 years, and in some studies, this percentage has been as high as 20%–30% (3). Many of these lesions are small when detected and guidelines for *in situ* lesions recommend either surgery alone or surgery followed by radiation as the usual treatment options. Treatment of DCIS is still exclusively determined by the extent and histological grade of the lesion (4). Our overall aim is to identify distinct subtypes of DCIS lesions that often progress to IBC, or not, to pave the way toward precision medicine for patients diagnosed with DCIS. We hypothesize that selected patients at high risk for the development of invasive cancers may require intensified, tailored, and targeted treatment, possibly including immunotherapy (e.g., anti-PD-1/PD-L1 (5, 6)) and anti-human epidermal growth factor receptor 2 (HER2) therapy, as offered to patients diagnosed with IBC (7–10). We believe that changing the current guidelines may not only have beneficial impacts on patients, avoiding under- and overtreatment, but also the health system will be able to better allocate funding to patients diagnosed with high-grade DCIS. To date, the utility of immunohistochemistry (IHC) markers has not been established in DCIS diagnostics, in contrast to IBC, where the hormone receptors (HRs) for estrogen (ER) and progesterone (PR), HER2, and Ki67 proliferation index are all deciding on a complex treatment algorithm. DCIS of the breast is a heterogeneous entity with nuclear atypia varying from mild to pronounced with various distinct growth patterns. We regularly confirm the presence of IHC-stained DCIS as part of the routine diagnosis of IBC but do not take much notice of it since these findings currently have no impact on therapy. Few studies have evaluated these markers in DCIS according to their molecular subtypes (11–13). In a previous study of high-grade breast DCIS, we reported the distribution of molecular subtypes. LumA and luminal B HER2-negative (LumB HER2^−^) cases together comprised 50.4%, luminal B HER2-positive (LumB HER2^+^) comprised 22.1%, HER2-enriched comprised 21.8%, and triple-negative (TPN) subtype comprised 5.6% of cases. We also identified the HER2-enriched subtype as a high-risk entity because it was significantly correlated with the presence of an invasive component (14, 15). This study aimed to investigate the pivotal and well-established molecular breast cancer markers, ER, PR, and HER2, in the respective subtypes of human high-grade DCIS, aiming to identify those DCIS lesions that most probably will progress to IBC.

## Materials and methods

Our study material consisted of formalin-fixed and paraffin-embedded (FFPE) histopathological specimens from the consecutive patient cohort stored in the diagnostic archive at Akershus University Hospital, Norway. Collected between 1996 and 2018, these samples represented 494 female patients diagnosed with DCIS of the breast. Experienced breast pathologists actively graded the histopathological specimens using the Van Nuys classification system (15, 16). To our knowledge, this is the largest DCIS biobank that has been approved for cancer research purposes in Europe. We chose to focus on and investigate grade 3 (high-grade) DCIS cases because these lesions are thought to have the highest risk of recurrence and progression to IBC (16–19). A total of 357 high-grade DCIS cases were submitted to IHC analysis, stained for ER, PR, HER2, and Ki67, and subjected to further studies. These were classified into their respective subtypes in accordance with the 2013 St. Gallen International Consensus Conference Guidelines, currently established for molecular subtyping of IBC lesions (15). Briefly, according to this classification (20), the LumA subtype was defined when ER was positive (≥ 1%) and/or PR was ≥ 20%, HER2^−^, and Ki67 index was < 20%. LumB HER2^−^ was defined as ER that was positive, HER2^−^, and Ki67 index expression was ≥ 20%, or when ER was ≥ 1%, Ki67 was ≥ 20% or PR expression was < 20%, and HER2 was negative. LumB HER2^+^ was defined when ER and/or PR were positive and HER2^+^ and Ki67 were at any value. HER2-enriched was defined as ER and PR negativity, HER2 positivity, and any Ki67 value. TPN was defined when ER, PR, and HER2 were negative and Ki67 was at any value. The general definitions of the molecular subtypes in IBC, according to IHC surrogate markers, are provided in **Supplementary Table S1** (21–23). All procedures have been described in detail in our previous study (15). Each subtype was sorted into three subcategories: “Pure” (*n* = 306) meaning those without an invasive component; “W/invasive” (*n* = 51) meaning those with an invasive component; and “All” (*n* = 357) meaning the entire group of the given subtype. We decided to split the patients based on age (younger than 50 and older than 50), taking into account other studies that looked at the incidence and mortality rate of DCIS (2, 24).

### Immunohistochemistry and dual-color silver-enhanced *in situ* hybridization

IHC staining for ER, PR, HER2, and Ki67 was performed using a Dako Autostainer (Agilent). Antigen retrieval was achieved in a PT-Link station by immersion in EnVision™ FLEX Target Retrieval Solution at a high pH (K8004, Agilent) and heating at 97°C for 20 min. Endogenous peroxidase activity was quenched by incubating the slides in the EnVision™ FLEX peroxidase blocking reagent (K8000, Agilent) for 5 min. For HER2 IHC, nonspecific staining was inhibited by an animal-free blocking solution 1× (No. 15019) for 30 min. Primary antibodies Ki67 (1:200), ER (1:50), and PR (1:100) were diluted in EnVision™ FLEX Antibody Diluent (K8006, Agilent); antibody HER2 (1:200) was diluted in SignalStain® Antibody Diluent (No. 8112, Cell Signaling), and slides were incubated with primary antibodies for 20–60 min at room temperature. For ER and PR IHC, rabbit (K800921–2, Agilent) and mouse (K800221–2, Agilent) linkers were added for 15 min for signal amplification after incubation with the primary antibody. This was followed by incubation with the ready‐to‐use secondary buffered solution (k8002, EnVision FLEX/HRP, Agilent) for 20 min. The sections were reacted with 3.30‐diamino‐benzidine tetrahydrochloride (DAB) solution for 10 min and counterstained with hematoxylin (link) (k8008, Agilent) for 5 min. In each run, a positive tissue control with invasive mammary carcinoma was included. Details of the antibody clones, staining, and dilutions are described in **Table 1**. The ER and PR IHC positivity was defined as ≥ 1% positive tumor (DCIS) cells in accordance with the updated guidelines of the American Society of Clinical Oncology (ASCO) and College of American Pathologists (CAP) (25) developed for IBC. We chose to divide the ER into the following intervals: < 1%, 1%–10%, > 10%–50%, and > 50%–100%, and PR in < 1%, 1%–20%, > 20%–50%, and > 50%–100%, based on St. Gallen 2013 and numerous other studies that examined the prognostic and predictive values in different HRs cutoff points (20, 26, 27). Ki67% IHC was estimated, by counting 200 DCIS cells in two separate hotspot foci, and the ratios were calculated and recorded as continuous values, rather than categorical values. Ki67 cutoff threshold was set at 20%, in accordance with the 2013 St. Gallen Recommendations (28) established for IBC. Three breast pathologists interpreted the IHC analysis in ER, PR, HER2, and Ki67. HER2 IHC was scored based on ASCO/CAP guidelines, as in routine diagnostics for IBC (29, 30). Briefly, HER2 was scored “0” when IHC staining was absent or membrane staining was weak and pale in ≤ 10% of DCIS cells. HER2 was considered “1+” when partial and incomplete membrane staining also showed a faint intensity within > 10% of the DCIS cells, and HER2 that was scored as “3+” showed strong and complete positive membrane staining in > 10% of DCIS cells. HER2 was identified as “2+” when the membrane was stained faint to moderately complete in > 10% of DCIS cells or strongly and completely in ≤ 10% of DCIS cells, which was considered equivocal and was subjected to further dual-color silver-enhanced *in situ* hybridization (dc-SISH) analysis performed on a Ventana BenchMark (Roche Diagnostics, Switzerland) machine using the fully automated Ultra-IHC/ISH Staining Module (31) with CC2 as a buffer. The dc-SISH results were interpreted in accordance with the ASCO/CAP comprehensive guidelines and algorithms established for IBC (32). We did not observe any changes in the quality or intensity of ER, PR, Ki67, HER2 IHC, or HER2 SISH, regardless of when the sample was taken.

**Table 1:** Details of antibodies’ clone, staining, and dilutions.

Antibody	Clone	Staining	Reference ID	Vendor	Dilution
**Anti-Ki67**	MIB-1	Nuclear	M724001–2	Agilent (USA)	1:200
**Anti-human estrogen receptor α**	EP1	Nuclear	M364301–2	Agilent (USA)	1:50
**Anti-human progesterone receptor**	PR 636	Nuclear	M356901–2	Agilent (USA)	1:100
**Anti-HER2/ErbB2**	D8F12	Membrane	#4290	Cell Signaling (USA)	1:200

### Statistical analysis

GraphPad Prism version 9.4 was used for the statistical calculations. We applied different tests to calculate statistical significance, ensuring they were conducted only when appropriate. Pearson’s Chi-square (*χ*²) or Fisher’s exact tests were used to calculate *p*-values when comparing two proportions, using the contingency table. Statistical significance was set *a priori* at *p* < 0.05.

## Results

### Hormone receptor status

**Figure 1:**
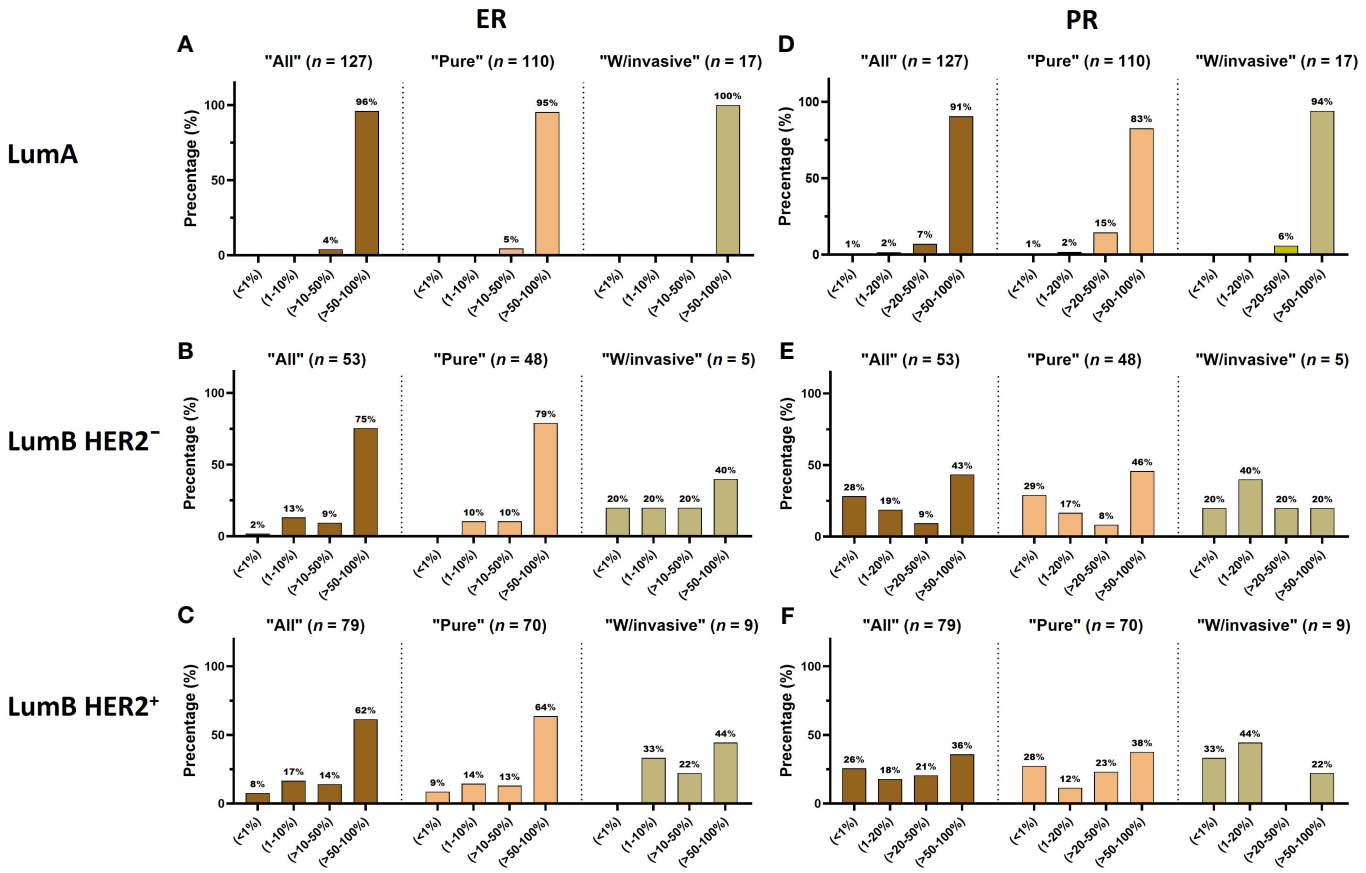
**(A–F)** The distribution of ER and PR expression is displayed in each luminal subtype (LumA, LumB HER2^−^ and LumB HER2^+^). Each subtype is further subcategorized into “All”, “Pure”, and “W/invasive”, respectively.

**Figure 2:**
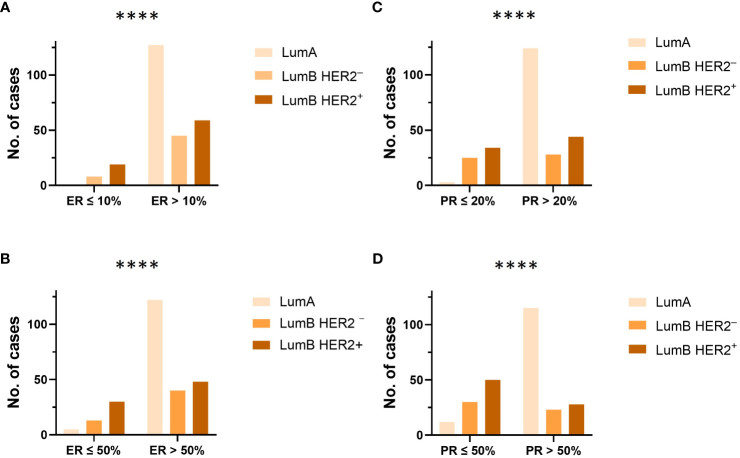
**(A, B)** The proportion and distribution of ER at cutoff values of 10% and 50%, were compared between each luminal subtype (LumA, LumB HER2^−^ and LumB HER2^+^), respectively. ^****^ *p*-value < 0.0001, Chi-square test. **(C, D)** The proportion and distribution of PR at cutoff values of 20% and 50%, were compared between each luminal subtype (LumA, LumB HER2^−^, and LumB HER2^+^), respectively. ^****^ *p*-value < 0.0001, Chi-square test.

**Figure 3:**
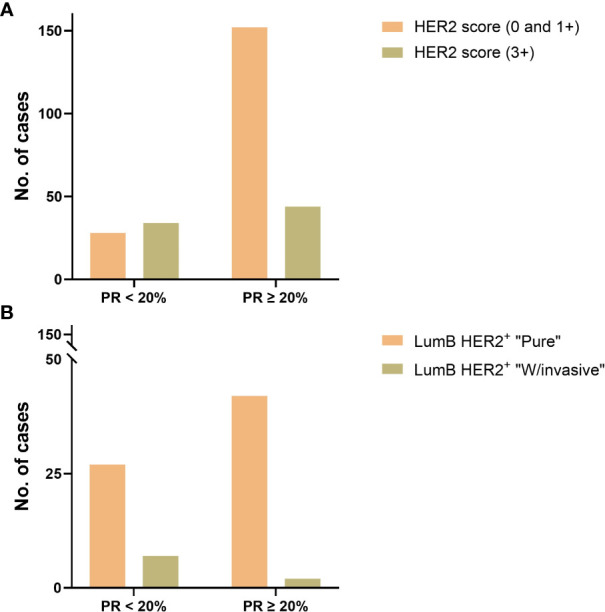
**(A)** PR expression status with a cutoff value of 20% was compared to HER2 IHC scores (“0”, “1+”, and “3+”) in luminal B subtypes and showed a significant difference (*p*-value = 0.0001, Fisher’s exact test) in favor of those with HER2 IHC score 3+. **(B)** PR expression status with a cutoff value of 20% in the LumB HER2+ subtype was compared in the “Pure” vs. “W/invasive” subcategories and showed a significant difference (*p*-value = 0.0365, Fisher’s exact test) in favor of the “W/invasive” component.

The majority of patients (71%) were older than 50 years (**Supplementary Figures S1A, B**; **Table 2**). There were no significant differences in the proportion of age in the “Pure” (*n* = 306) versus “W/invasive” (*n* = 51) groups. Of “All” cases, 98% of the LumA subtype showed an ER ≥ 50%. PR expression ≥ 50% was found in 91% of cases in this subtype. In general, the expression of PR was slightly lower than that of ER in the LumA subtype. Details of ER and PR expression in luminal subtypes (LumA, LumB HER2^−^, and LumB HER2^+^) according to 2013 St. Gallen consensus meeting guidelines are shown in **Figures 1A-F**; **Supplementary Table S2**. The incidence of ER-positive cases at a cutoff of < 10% in the LumA subtype was significantly lower than that in the LumB HER2^−^ and LumB HER2^+^ subtypes (*p* < 0.0001, chi-square) (**Figure 2A**). In contrast, there was a statistically significant increase in ER expression at a cutoff of ≥ 50% in the LumA compared to the latter subtypes (*p* < 0.0001, chi-square) (**Figure 2B**). The proportion of PR-positive cases at a cutoff of < 20% showed significant differences in luminal subtypes between the LumA, LumB HER2^−^, and LumB HER2^+^ subtypes (3%, 47%, and 44%, respectively) (*p* < 0.0001, Chi-square) (**Figure 2C**). There was also a significantly higher proportion of patients with a PR ≥ 50% among the LumA subtype (*p* < 0.0001, Chi-square) (**Figure 2D**). We demonstrated that low PR expression (< 20%) was significantly associated with a concurrent invasive component (*p* = 0.0365, Fisher’s exact test) (**Figure 3A**). We observed no significant differences in ER expression (< 1%, 1%–10%, > 10%–50%, > 50%–100%) and PR expression (< 1%, 1%–20%, > 20%–50%, > 50%–100%) between luminal subtypes in “Pure” cases of high-grade DCIS and those “W/invasive” components.

**Table 2:** Distribution of age ≶ 50 years is demonstrated among all subtypes that are further subcategorized in “Pure” and “W/invasive”, respectively.

Age (years)	LumA “Pure” (*n* = 110)	LumB HER2^−^ “Pure” (*n* = 48)	LumB HER2^+^ “Pure” (*n* = 70)	HER2-enriched “Pure” (*n* = 60)	TPN “Pure” (*n* = 18)
< 50	34%	31%	31%	23%	6%
≥ 50	66%	69%	69%	77%	94%
**Age (years)**	**LumA** **“W/invasive”** (*n* = 17)	**LumB HER2** ^−^ **“W/invasive”** (*n* = 5)	**LumB HER2^+^** **“W/invasive”** (*n* = 9)	**HER2-enriched** **“W/invasive”** (*n* = 18)	**TPN** **“W/invasive”** (*n* = 2)
< 50	35%	20%	56%	22%	50%
≥ 50	65%	80%	44%	78%	50%

### HER2 status

**Figure 4:**
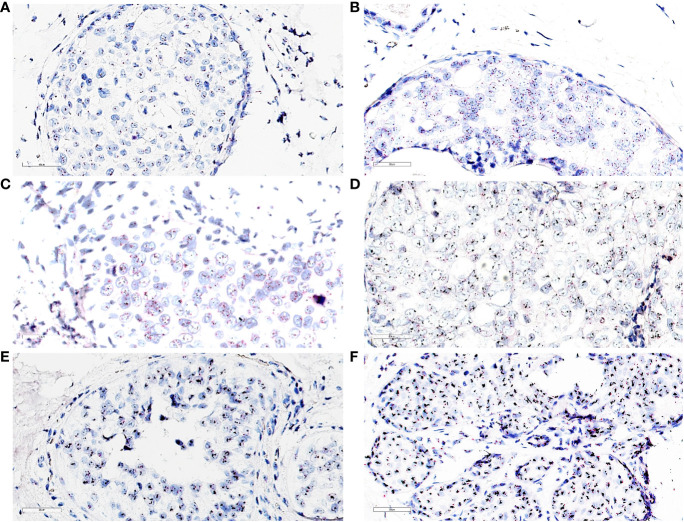
**(A)** dc-SISH of HER2 (black signals) and CEP17 (red signals) displaying nonamplification (disomy). HER2/CEP17 was equal to 1.25 (2.75/2.2); ×40 magnification. **(B)** dc-SISH of HER2 (black signals) and CEP17 (red signals) displaying a heterogenous nonamplification with two to four HER2 and CEP17 signals. HER2/CEP17 was equal to 1.16 (3.43/2.95); ×40 magnification. **(C)** dc-SISH of HER2 (black signals) and CEP17 (red signals) displaying tetrasomy nonamplification. HER2/CEP17 was equal to 1.16 (4.65/4.0); ×40 magnification. **(D)** dc-SISH of HER2 (black signals) and CEP17 (red signals) displaying amplification. HER2/CEP17 was equal to 4.67 (7.7/1.65); ×40 magnification. **(E)** dc-SISH of HER2 (black signals) and CEP17 (red signals) displaying HER2/CEP17 high amplification (cluster); ×40 magnification. **(F)** dc-SISH of HER2 (black signals) and CEP17 (red signals) displaying HER2/CEP17 high amplification (cluster) with retrograde lobular cancerization (*cancerization* of lobules) growth pattern; ×40 magnification.

The distribution of HER2 IHC scores showed the proportions for score 0 as 41%; score 1+ as 13%; score 2+ as 4%; and score 3+ as 42%. Of the LumA cases, 96% were HER2^−^, with an IHC score of 0 or 1+ (**Table 3**). We compared the HER2 IHC 0, 1+, and 2+ scores among LumA and LumB HER2^−^ subtypes and did not find statistically significant differences when the “Pure” and “W/invasive” were compared (*p* = 0.603, Chi-square). We found a significant association between HER2 overexpression (score 3+) and low PR expression (< 20%) in the luminal B subtypes (*p* = 0.0001, Fisher’s exact test) (**Figure 3B**). A total of 16 cases were HER2 IHC equivocal (2+ score) and subjected to HER2 dc-SISH analysis. Five cases belonged to LumA, one to LumB HER2^−^, seven to LumB HER2^+^, and one to HER2-enriched, whereas two samples belonged to the TPN subtype. Nonamplified cases had a mean of 3.1 HER2 gene signals and 2.3 chromosome 17 (CEP17) signals. Amplification by dc-SISH was observed in 50% of cases. The LumB HER2^+^ subtype accounted for 88% of all dc-SISH amplification cases. **Figures 4A-F** display dc-SISH staining in selected cases, from nonamplification to high amplification (clusters). Details of the dc-SISH findings, HER2 gene, and centromeric probe for CEP17 count and ratios are summarized in **Table 4**.

**Table 3:** HER2 IHC expression is displayed according to the molecular subtypes.

Subtypes	0 (*n* = 147; 41%)	1+ (*n* = 45; 13%)	2+ (*n* = 16; 4%)	3+ (*n* = 149; 42%)	Subtotal (*n* = 357; 100%)
**LumA** (*n* = 127)	97 (76%)	25 (20%)	5 (4%)		*n* = 127
“Pure” (*n* = 110)	85 (77%)	20 (18%)	5 (5%)		
“W/invasive” (*n* = 17)	12 (70%)	5 (30%)			
**LumB HER2** ^−^ (*n* = 53)	37 (70%)	15 (28%)	1 (2%)		*n* = 53
“Pure” (*n* = 48)	34 (71%)	13 (27%)	1 (2%)		
“W/invasive” (*n* = 5)	3 (60%)	2 (40%)			
**LumB HER2^+^** (*n* = 79)			7 (9%)	72 (91%)	*n* = 79
“Pure” (*n* = 70)			6 (9%)	64 (91%)	
“W/invasive” (*n* = 9)			1 (11%)	8 (89%)	
**HER2-enriched** (*n* = 78)			1 (1%)	77 (99%)	*n* = 78
“Pure” (*n* = 60)			1 (2%)	59 (98%)	
“W/invasive” (*n* = 18)				18 (100%)	
**TPN** (*n* = 20)	13 (65%)	5 (25%)	2 (10%)		*n* = 20
“Pure” (*n* = 18)	12 (67%)	4 (22%)	2 (11%)		
“W/invasive” (*n* = 2)	1 (50%)	1 (50%)			

The number (n) of HER2 IHC score (“0”, “1+”, “2+”, and “3+”) is given for each respective subtype. The proportion of the HER2 IHC score is given within each subtype.

**Table 4:** The details of dc-SISH analyses on HER2 2+ (IHC) samples.

Subtype	HER2 signals	CEP17 signals	HER2-CEP17 ratio	Amplified?
**LumA**	3.2	2.45	1.3	No
**LumA**	2.14	1.64	1.3	No
**LumA**	2.0	2.0	1.0	No
**LumA**	2.9	2.25	1.29	No
**LumA**	3.95	3.2	1.23	No
**LumB HER2** ^−^	3.43	2.95	1.16	No
**LumB HER2^+^**	Clusters			Yes
**LumB HER2^+^**	Clusters			Yes
**LumB HER2^+^**	Clusters			Yes
**LumB HER2^+^**	Clusters			Yes
**LumB HER2^+^**	7.7	1.6	4.7	Yes
**LumB HER2^+^**	7.5	1.7	4.4	Yes
**LumB HER2^+^**	10.7	2.6	4.1	Yes
**HER2-enriched**	8.2	1.5	5.5	Yes
**TPN**	4.6	4.0	1.1	No
**TPN**	2.7	2.2	1.2	No

## Discussion

ER regulates PR expression in human breast tissue; thus, the latter hormone receptor is a clinical prognostic marker of ER action (33, 34). Therefore, low PR expression may indicate low or fading ER expression. Overall, our results demonstrated considerable heterogeneity in high-grade DCIS. High ER and PR expression were significantly more prevalent in the LumA subtype than in the LumB HER2^−^ and LumB HER2^+^ subtypes. In contrast, low ER and PR expression were more common in the latter two subtypes (*p* < 0.0001, Chi-square). Our results are consistent with those made in IBC (23). Furthermore, we found that low PR expression was significantly associated with HER2 overexpression (IHC score 3+) in both luminal B subtypes and in those with an invasive component (*p* = 0.0001 and *p* = 0.0365, respectively; Fisher’s exact test). These findings indicate that low PR expression in DCIS is an independent marker for progression to IBC and upregulation of the HER2 gene, with poor outcomes in patients diagnosed with IBC (35–39). Konecny et al. (40) identified an inverse relationship between HR levels and HER2 gene amplification in a large number of human breast cancer tissues. Huang HJ et al. investigated the relationship between age, HRs, and HER2 status in female patients with invasive breast cancer (41). They found that the relationship between HRs and HER2 expression varied with patient age, with a negative correlation primarily observed in patients aged > 45 years. This is an intriguing finding given that the majority (71%) of patients in our study material were over the age of 50 years (**Supplementary Figures S1A, B**). Shah et al. (42) reported a significant difference between age and ER/PR expression, although they did not identify any significant variation between age and HER2 overexpression. In our earlier study, we identified that HER2 overexpression in cases classified as the HER2-enriched subtype was concurrent with early morphological invasive growth in high-grade DCIS (15). HER2 gene amplification in IBC is an independent poor prognostic factor (43, 44). The prognostic and predictive roles of PR in IBC have been reported by others in some previous studies (35, 36, 45–47). However, while drugs that target ER are common therapeutic tools for the treatment of patients diagnosed with IBC (48), an effective drug targeting PR has not yet been approved for the treatment of these patients (35). Further studies are necessary to investigate whether, in diagnosing IBC and DCIS, PR can possibly be a reliable and reproducible prognostic and predictive marker in selected patients. Our results showed that HER2 expression varied greatly, with no significant differences between the LumA and LumB HER2^−^ subtypes (**Table 3**). The LumB HER2^+^ subtype had the highest number of equivocal (IHC 2+ score) lesions (7 of 16) and harbored an IHC 3+ score in 72 of 149 (48%) cases. The HER2-enriched subtype was uniform and had a strong IHC 3+ score in all except one case, whereas the LumB HER2^+^ subtype was quite heterogeneous, showing cluster amplification as well as polysomy and aneusomy with varying HER2 ratios, consistent with a lower grade of HER2 gene amplification. Our findings show that LumB subtypes are a heterogeneous family that displays a biological continuum of alterations in growth pattern, HR, and HER2 expression. Bediaga et al. (49) demonstrated that, in IBC, the LumB HER2 subtypes (HER2^+^ and HER2^−^) had both low and high DNA methylation categories, resulting in different epigenetic and clinical features. The high DNA methylation subgroup corresponded to the LumB HER2^+^ subtype, whereas the low DNA methylation group clustered with the LumA subtype. Our results also support this finding since only the present cutoff value for the Ki67 proliferation index determines whether a DCIS sample that is HR-positive and HER2^−^, is categorized as LumA or LumB HER2^−^. In the context of IBC, these cutoff values have been subject to discussions and changes in numerous studies (28, 50, 51). We investigated ER and PR expression in DCIS with various cutoff values and did not demonstrate any statistically significant differences between cases classified as “Pure” with those classified as “W/invasive”.

### Strengths and limitations of the study

To our knowledge, this study material is the largest DCIS biobank that has been approved for cancer research purposes in Norway. It is a large collection of tissue material that represents a 22-year period and was obtained during the initial diagnosis. All samples were assessed and rated by qualified mammary pathologists. IHC analyses were performed in a single pathology department in accordance with national standards and recommendations.

### Limitations of the study

Pathologists and clinicians are aware of the challenges associated with the Ki67 IHC assessment, which can lead to differences in the results of analyses within and between departments of pathology.

## Conclusions

The majority of patients (71%) in our material study were over 50 years old. IHC analysis of ER and PR revealed a spectrum from low to high expression within the DCIS LumA and LumB subtypes, with the former displaying significantly higher expression than the latter (*p* < 0.0001). According to our results, the LumB subtypes are a diverse group that exhibits a biological continuum of changes in HR, growth pattern, and HER2 amplification. In this study, we demonstrated that low PR in high-grade DCIS was linked to concurrent HER2 overexpression as well as the coexistence of an invasive component in the luminal B subtypes (*p* = 0.0001 and *p* = 0.0365, respectively). PR and HER2 may have the potential to be incorporated into diagnostic tools as robust and reproducible prognostic and predictive markers for distinguishing high-risk *in situ* lesions that progress to IBC. The results of this study may contribute to the identification of high-risk patients with DCIS potentially in need of systemic adjuvant therapy. This question has to be addressed in clinical trials.

## Data availability statement

The datasets for this article are not publicly available due to concerns regarding participant/patient anonymity. Requests to access the datasets should be directed to the corresponding author.

## Ethics statement

The studies involving humans were approved by Regional Committee for Medical and Health Research Ethics (REK). The studies were conducted in accordance with the local legislation and institutional requirements. Written informed consent for participation was not required from the participants or the participants’ legal guardians/next of kin because all living patients received an information letter in which the purpose of the project was described. The text of the information letter was approved by the Regional Committee for Medical and Health Research Ethics (REK) (case Nos. 29299 and 307976). A prepaid return envelope was enclosed in the letters patients received, in addition to a sheet to sign and return if they objected. If they agreed, they would not need to undertake any action. We received reservations from ten patients; consequently, their cases were excluded from further examinations.

## Author contributions

HS: Conceptualization, Data curation, Formal analysis, Investigation, Methodology, Project administration, Resources, Software, Validation, Visualization, Writing – original draft, Writing – review & editing. LF: Conceptualization, Methodology, Supervision, Validation, Writing – review & editing. DP: Formal analysis, Validation, Writing – review & editing. YL: Visualization, Writing – review & editing. SA: Formal analysis, Methodology, Writing – review & editing. JG: Conceptualization, Formal analysis, Funding acquisition, Methodology, Project administration, Resources, Supervision, Validation, Visualization, Writing – review & editing. TS: Conceptualization, Data curation, Formal analysis, Investigation, Project administration, Supervision, Validation, Visualization, Writing – review & editing.
