# Supplementary material for: Low progesterone receptor levels in high-grade DCIS correlate with HER2 upregulation and the presence of invasive components
Source: Front Oncol. 2024 Jun 26;14:1347166. doi: 10.3389/fonc.2024.1347166 (PMC11247389; doi:10.3389/fonc.2024.1347166)
Supplement: Supplementary file 1 [file DataSheet_1.docx]

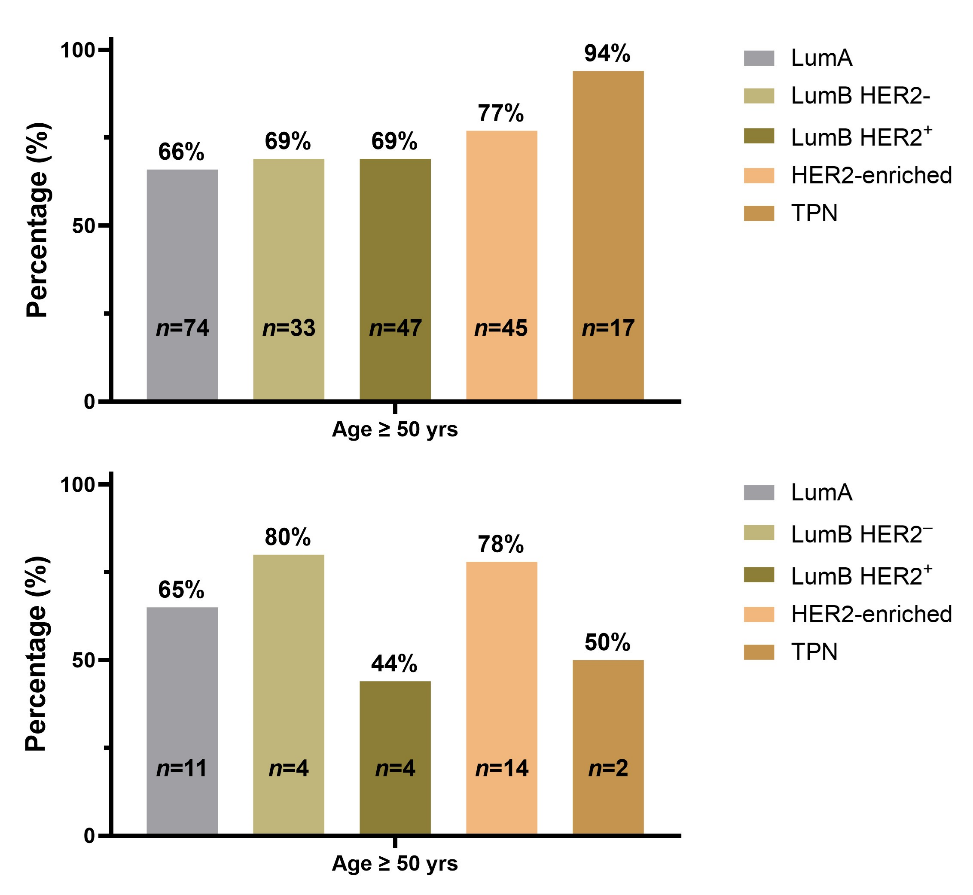
**Supplementary Figure 1a-b** Percentage and number of patients ≥ 50 yrs old, in respective subtypes and subcategorized in “pure” (**2a**) and “w/invasive” (**2b**), respectively

**Supplementary Table 1.** Definition of molecular subtypes for invasive breast carcinoma is given according to surrogate immunohistochemistry markers

| **LumA** | Hormone receptors (ER and/or PR) positive, HER2-negative, a low level of proliferation according to the Ki67 proliferation index. Luminal A tumors are low-grade, tend to grow slowly, and have the best prognosis |
| --- | --- |
| **LumB HER2ˉ** | Hormone receptors (ER and/or PR) positive, HER2-negative, and with higher levels of Ki67 proliferation index, than luminal A tumors |
| **LumB HER2^+^** | Hormone receptors (ER and/or PR) positive, HER2-positive, and any value level of Ki67 proliferation index |
| **HER2-enriched** | Hormone receptors (ER and PR) negative, HER2-positive, and any value level of Ki67 proliferation index |
| **TPN** | Hormone receptors (ER and PR) and HER2-negative, any value level of Ki67 proliferation index |

**Supplementary Table 2.** Distribution of ER and PR expression is shown among LumA, LumB HER2ˉ and LumB HER2^+^ subtypes, respectively

| **Subtypes** | **ER (<1%)** | **ER (1 - 10%)** | **ER (>10 - 50%)** | **ER (>50 - 100%)** |  | **PR (<1%)** | **PR (1 - 20%)** | **PR** **(>20 - 50%)** | **PR (>50 - 100%)** |
| --- | --- | --- | --- | --- | --- | --- | --- | --- | --- |
| **LumA** “All” *n* = 127 |  |  | 5  (4%) | 122  (96%) |  | 1 (1%) | 2 (2%) | 9 (7%) | 115 (90%) |
| “Pure” *n* = 110 |  |  | 5  (5%) | 105 (95%) |  | 1 (1%) | 2 (2%) | 16  (14%) | 91 (83%) |
| “W/invasive component” *n* = 17 |  |  |  | 17 (100%) |  |  |  | 1 (6%) | 16 (94%) |
| **LumB HER2ˉ** “All” n = 53 | 1 (2%) | 7  (13%) | 5 (9%) | 40 (76%) |  | 15 (28%) | 10 (19%) | 5  (10%) | 23 (43%) |
| “Pure” *n* = 48 |  | 5  (10%) | 5 (10%) | 38 (80%) |  | 14  (29%) | 8 (17%) | 4 (8%) | 22 (46%) |
| “W/invasive component” *n* = 5 | 1  (20%) | 1  (20%) | 1 (20%) | 2  (40%) |  | 1 (20%) | 2 (40%) | 1 (20%) | 1 (20%) |
| **LumB HER2^+^** “All” *n* = 79* | 6 (8%) | 13 (17%) | 11 (14%) | 48 (61%) |  | 20 (26%) | 14 (18%) | 16 (20%) | 28 (36%) |
| “Pure” *n* = 70* *one sample lacked ER | 6  (9%) | 10  (14%) | 9 (13%) | 44 (64%) |  | 19 (28%) | 8 (12%) | 16 (23%) | 26 (37%) |
| “W/invasive component” *n* = 9 |  | 3  (33%) | 2 (22%) | 4 (45%) |  | 3  (33%) | 4 (44%) |  | 2  (22%) |
